# Compliance With Triage Protocols in the Emergency Department: A Retrospective Audit

**DOI:** 10.7759/cureus.100986

**Published:** 2026-01-07

**Authors:** Fatima Amjad, Baseera Imran, Affia Altaf, Mansoor Ul Hassan, Sana Qadeer, Qudsiah Ghazanfar, Furqan Mushtaq

**Affiliations:** 1 Internal Medicine, Shalamar Hospital, Lahore, PAK; 2 Physiology, Ameer-ud-Din Medical College, Lahore, PAK; 3 Internal Medicine, Combined Military Hospital, Multan, PAK; 4 Internal Medicine, Holy Family Hospital, Rawalpindi, PAK; 5 Internal Medicine, Rawalpindi Medical University, Rawalpindi, PAK; 6 Intensive Care Unit, Shalamar Hospital, Lahore, PAK

**Keywords:** australasian triage scale, canadian triage and acuity scale, emergency department, emergency severity index, manchester triage system, triage protocols

## Abstract

Background

Triage is a critical component of emergency department (ED) operations, ensuring that patients are prioritized according to the severity of their condition.

Objective

This study aims to evaluate compliance with standardized triage protocols in a tertiary care ED and to determine the frequency of under-triage and over-triage in relation to patient characteristics and time of presentation.

Materials and methods

This retrospective observational study was conducted at Shalamar Hospital, Lahore, Pakistan, from March 2022 to March 2025. Medical records of 135 patients presenting to the ED during the study period were reviewed. Data collected included demographics, presenting complaints, assigned triage categories, and compliance with institutional triage guidelines. Compliance was defined as adherence to standardized triage criteria, while deviations were categorized as under-triage or over-triage. Associations between compliance and patient characteristics were assessed using Pearson’s chi-square test, with p < 0.05 considered statistically significant.

Results

The mean age of patients was 42.6 ± 18.3 years, with 83 (61.5%) males and 52 (38.5%) females. Medical presentations were most frequent (62.2%), followed by surgical (23.7%) and trauma-related (14.1%) cases. Overall compliance with triage protocols was 75.6% (102/135 cases), while 24.4% (33/135 cases) were non-compliant. Among non-compliant cases, 21 (15.6%) were under-triaged and 12 (8.9%) over-triaged. Compliance was highest among Category I patients (86.4%) and lowest among Category III patients (69.2%). Significant associations were observed between compliance and both triage category (χ² = 5.56, p = 0.04) and time of presentation (χ² = 4.71, p = 0.03), with lower compliance during peak hours.

Conclusions

Compliance with triage protocols in the ED was moderately high but suboptimal, with nearly one-fourth of patients triaged incorrectly. Under-triage was more common, representing a significant safety concern. Compliance was significantly influenced by triage category and time of presentation, highlighting the need for staff training, system support, and workload management to improve triage accuracy.

## Introduction

Triage is a fundamental concept in emergency medicine, serving as the initial decision-making process in patient care within the emergency department (ED). The term “triage” is derived from the French verb trier, meaning “to sort” [1], and reflects the primary objective of categorizing patients according to the urgency of their clinical condition. This ensures that patients with life-threatening conditions receive immediate attention, while those with less urgent needs are managed in a timely and resource-conscious manner [2].

With rising patient volumes globally, triage has become increasingly critical for patient safety, efficient resource utilization, and equitable access to emergency care [3]. To standardize this process, structured triage systems have been developed and widely implemented, including the Emergency Severity Index (ESI), the Manchester Triage System (MTS), the Canadian Triage and Acuity Scale (CTAS), and the Australasian Triage Scale (ATS). These systems use objective criteria, such as presenting symptoms, vital signs, and risk factors, to assign patients to acuity levels that reflect the urgency of care required and anticipated resource utilization [4]. When applied correctly, structured triage systems reduce subjectivity, improve clinical performance, and enhance multidisciplinary communication within the ED [5].

However, the effectiveness of these systems is highly dependent on compliance. Even the most robust triage tool is ineffective if not implemented consistently and accurately by healthcare staff. Non-compliance, whether due to lack of knowledge, time pressures, overcrowding, or systemic inefficiencies, undermines the purpose of triage and creates variability in patient care [6]. Under-triage, in which a patient’s acuity is underestimated, can delay critical interventions and increase morbidity and mortality. Conversely, over-triage leads to unnecessary resource utilization and contributes to ED overcrowding, which is associated with worse patient outcomes and staff burnout. Thus, adherence to triage protocols is not merely a technical issue but a crucial patient safety concern [7].

The complexity of ED environments further complicates compliance. EDs face unpredictable, fluctuating patient inflows, ranging from trauma cases to outbreaks or mass-casualty incidents [8]. Factors such as staff-to-patient ratio, triage staff training, clinical experience, and institutional support all influence adherence levels [9]. In low- and middle-income countries, additional challenges, such as limited staffing, resource constraints, and inadequate training infrastructure, contribute to high variability in triage outcomes. Worldwide ED overcrowding underscores the need for effective triage compliance [10]. Given the mounting pressures on healthcare systems, aging populations, and episodic crises such as pandemics, the role of triage in ensuring efficient and safe emergency care cannot be overestimated [11].

Evidence indicates that adherence to triage protocols is directly associated with reduced waiting times, improved patient satisfaction, and enhanced workflow efficiency among ED staff, whereas non-adherence results in inefficiency, staff frustration, and substandard care [12].

This study aimed to evaluate compliance with standardized triage protocols in a tertiary care ED and to determine the frequency of under-triage and over-triage in relation to patient characteristics and time of presentation.

## Materials and methods

Study design and setting

This study was a retrospective observational audit conducted in the ED at Shalamar Hospital, Lahore, Pakistan, a tertiary care teaching hospital that provides 24-hour emergency services. The ED receives approximately 70,000-90,000 patient visits annually and follows a standardized triage protocol based on internationally recognized triage systems. Initial triage is routinely performed by trained triage nurses at the point of first patient contact, in accordance with institutional policy.

The audit was carried out between March 2022 and March 2025 and included all eligible cases documented during the study period. A total of 135 patient records were selected using systematic sampling, in which every nth eligible record from the ED registry during the study period was included to ensure representation across days, months, and shifts. The purpose of this audit was to assess compliance with institutional triage procedures and identify deviations that could affect patient safety and clinical workflow.

Triage system and tools used

The hospital’s triage protocol is adapted primarily from ESI version 4, a widely used, free-to-use triage tool provided by the U.S. Agency for Healthcare Research and Quality [13]. No licensing fees or formal permissions were required for its use in clinical practice or academic review, as ESI is available in the public domain.

In certain clinical scenarios where additional guidance was needed (e.g., trauma or mixed clinical presentations), components of the MTS were referenced at the departmental level; however, the MTS algorithm is a licensed tool owned by the Manchester Triage Group (MTG) [14]. MTS is incorporated institutionally through authorized training modules available to hospital triage nurses. No proprietary MTS materials were reproduced or analyzed in this study; thus, no formal licensing approval was required for this retrospective audit.

Inclusion and exclusion criteria

All patients, irrespective of age or gender, who presented to the ED and had documented triage assessments performed by triage staff during the study period were included; patients with incomplete triage records, those directly admitted to inpatient wards or intensive care units without undergoing a formal triage process, and cases in which triage documentation was illegible or missing essential parameters were excluded.

Data collection

Data were collected retrospectively from ED medical records and triage forms. A structured proforma was used to standardize data abstraction. The variables collected included demographic information (age, sex), presenting complaints, vital signs at presentation, triage category, time of arrival, and eventual management decision (admission, transfer, or discharge).

Triage decisions documented in patient records were compared with the hospital’s standardized triage protocols (e.g., ESI, MTS) to assess compliance. Non-compliance was defined as any deviation from the established triage criteria, including assigning an incorrect triage category or failing to record essential parameters required for accurate classification.

Assessment of compliance

Two independent reviewers, both ED physicians with triage experience, evaluated each record. Reviewers were not involved in the original triage decisions and underwent a calibration exercise before data review to standardize the interpretation of triage criteria. Any discrepancy was resolved through consensus discussion to minimize subjective bias. Inter-rater agreement was assessed using Cohen’s kappa statistic to ensure the reliability of the compliance assessment.

Deviations were classified as (a) under-triage, where a patient is assigned a lower acuity level than required, and (b) over-triage, where a patient is assigned a higher acuity level than required.

The presence of missing vital signs or essential triage parameters was also recorded as non-compliance. Cases with missing key variables that precluded accurate reassessment were excluded from the final analysis.

Data analysis

Data were entered and analyzed using SPSS Statistics version 26.0 (IBM Corp., Released 2019. IBM SPSS Statistics for Windows, Version 26.0. Armonk, NY: IBM Corp.). Continuous variables, such as patient age, were expressed as mean ± standard deviation (SD), whereas categorical variables, including gender, presenting complaints, and triage categories, were reported as frequencies and percentages. Compliance rates were calculated as the proportion of cases adhering to institutional triage protocols. Associations between compliance and patient characteristics (e.g., age, gender, presenting complaints, time of arrival) were evaluated using Pearson’s chi-square test. Inter-rater reliability was analyzed using Cohen’s kappa coefficient. A p-value < 0.05 was considered statistically significant. Missing data were handled through case-wise deletion, and sensitivity analysis was performed to ensure that excluded records did not significantly bias the results.

## Results

A total of 135 patient records were analyzed, with a mean age of 42.6 ± 18.3 years. The majority of patients were male (83/135, 61.5%), while females comprised 52/135 (38.5%). Most patients presented with medical complaints (84/135, 62.2%), followed by surgical cases (32/135, 23.7%) and trauma-related emergencies (19/135, 14.1%). Regarding triage assignment, 22/135 patients (16.3%) were classified as Category I (critical), 48/135 (35.6%) as Category II (urgent), and 65/135 (48.1%) as Category III (stable). Overall compliance with triage protocols was observed in 102/135 cases (75.6%), whereas 33/135 cases (24.4%) were non-compliant. Among the non-compliant cases, under-triage occurred more frequently (21/135, 15.6%) than over-triage (12/135, 8.9%) (Table 1).

**Table 1:** Baseline demographic and clinical characteristics of patients (n = 135) Data are presented as N (%) or mean ± SD. Percentages are calculated for the total population. * According to ESI/MTG [13,14] SD: standard deviation, ESI: Emergency Severity Index, MTG: Manchester Triage System

Characteristic	Value
Age (years), mean ± SD	42.6 ± 18.3
Gender, n (%)	
Male	83 (61.5%)
Female	52 (38.5%)
Type of presentation, n (%)	
Medical	84 (62.2%)
Surgical	32 (23.7%)
Trauma	19 (14.1%)
Triage category assigned*, n (%)	
Category I (critical)	22 (16.3%)
Category II (urgent)	48 (35.6%)
Category III (stable)	65 (48.1%)
Compliance status, n (%)	
Compliant	102 (75.6%)
Non-compliant	33 (24.4%)
Under-triage	21 (15.6%)
Over-triage	12 (8.9%)

When compliance was stratified by triage category, Category I patients had the highest compliance rate, with 19/22 cases (86.4%) accurately triaged, followed by Category II at 37/48 cases (77.1%). The lowest compliance was observed in Category III, where 20/65 cases (30.8%) were non-compliant. These findings indicate that deviations from protocol were more frequent among patients with less acute presentations (Table 2).

**Table 2:** Compliance by triage category (n = 135) Data are presented as N (%). Associations between compliance and triage category were assessed using Pearson’s chi-square test. χ² and p-values refer to the overall comparison for the variable. Significant at p < 0.05. * According to the ESI/MTG [13,14] ESI: Emergency Severity Index, MTG: Manchester Triage Group

Variable	Category/time	Total (n)	Compliant n (%)	Non-compliant n (%)	χ² value	p-value
Triage category*	Category I (critical)	22	19 (86.4%)	3 (13.6%)	5.56	0.04*
	Category II (urgent)	48	37 (77.1%)	11 (22.9%)		
	Category III (stable)	65	45 (69.2%)	20 (30.8%)		

Compliance did not differ significantly between genders. Among males, 65/83 cases (78.3%) were compliant, compared to 37/52 cases (71.2%) among females (χ² = 0.83, p = 0.36). Similarly, the type of presentation was not significantly associated with compliance (χ² = 3.16, p = 0.21). Compliance rates were 67/84 (79.8%) for medical cases, 23/32 (71.9%) for surgical cases, and 12/19 (63.2%) for trauma cases. These findings suggest that neither gender nor type of presentation significantly influenced adherence to triage protocols (Table 3).

**Table 3:** Compliance by gender and type of presentation (n = 135) Data are presented as N (%). Associations between compliance and categorical variables were assessed using Pearson’s chi-square test. χ² and p-values refer to the overall comparison for the variable. Significant at p < 0.05.

Variable	Category/type	Total (n)	Compliant n (%)	Non-compliant n (%)	χ² (overall)	p-value (overall)
Gender	Male	83	65 (78.3%)	18 (21.7%)	0.83	0.36
	Female	52	37 (71.2%)	15 (28.8%)		
Type of presentation	Medical	84	67 (79.8%)	17 (20.2%)	3.16	0.21
	Surgical	32	23 (71.9%)	9 (28.1%)		
	Trauma	19	12 (63.2%)	7 (36.8%)		

Compliance varied significantly according to the time of presentation. During peak hours (6 pm-12 am), 44/66 cases (66.7%) were compliant, whereas compliance was higher during off-peak hours (12 am-6 pm) at 58/69 cases (84.1%) (χ² = 4.71, p = 0.03). This indicates that adherence to the triage protocol was lower during busier periods in the ED (Table 4).

**Table 4:** Compliance by time of presentation (n = 135) Data are presented as N (%). Associations between compliance and time of presentation were assessed using Pearson’s chi-square test. χ² and p-value refer to the overall comparison for the variable. Significant at p < 0.05.

Time of presentation	Total (n)	Compliant n (%)	Non-compliant n (%)	χ² (overall)	p-value (overall)
Peak hours (6 pm–12 am)	66	44 (66.7%)	22 (33.3%)	4.71	0.03*
Off-peak hours (12 am–6 pm)	69	58 (84.1%)	11 (15.9%)		

## Discussion

The present study is a retrospective analysis evaluating adherence to triage procedures in the ED, based on a review of 135 patient charts. Overall compliance with triage protocols was 102/135 cases (75.6%), indicating that the majority of assessments followed established guidelines, although approximately one-fourth of cases deviated. Among non-compliant cases, under-triage occurred in 21/135 cases (15.6%), while over-triage was observed in 12/135 cases (8.9%). Under-triage is particularly concerning, as it delays timely intervention for patients with potentially life-threatening conditions.

Compliance analysis by triage category revealed the highest adherence in Category I (critical) patients, with 19/22 cases (86.4%) accurately triaged. This suggests that triage staff are more vigilant in identifying severely ill patients. However, compliance declined with lower-acuity patients, reaching its lowest in Category III (stable) patients at 45/65 cases (69.2%). This trend aligns with prior studies emphasizing increased variability and subjectivity in the assessment of stable patients, whose symptoms are less pronounced and resource needs are less predictable [15]. These findings reinforce the importance of accurate triage across all patient acuity levels, not only for critically ill patients.

Non-compliance was also significantly associated with the time of presentation, being more frequent during peak hours (6 pm-12 am) with 22/66 cases non-compliant (33.3%) compared to off-peak hours (12 am-6 am) at 11/69 cases (15.9%) (χ² = 4.71, p = 0.03). This reflects the challenges of ED overcrowding and staff fatigue, which may reduce adherence to standardized protocols. While the overall compliance rate in this study is relatively high, it remains suboptimal. In high-resource settings, compliance rates often exceed 85%, supported by electronic decision-support systems, frequent audits, and structured training programs [16].

These findings have important implications for patient safety and ED efficiency. Under-triage can delay critical interventions, increasing morbidity and mortality, whereas over-triage, although less harmful to individual patients, can strain resources and contribute to overcrowding. Both deviations compromise the core objectives of triage: ensuring safety, efficiency, and equitable distribution of emergency care [17]. Addressing these issues requires a multi-level approach, including routine training, simulated exercises to enhance staff confidence in protocol implementation, regular audits to provide feedback and accountability, and integration of digital triage support systems to reduce subjectivity and aid decision-making under stress [18-20].

This study has several limitations. Being retrospective, it relied on the completeness and accuracy of documentation, which may not fully reflect real-time triage decision-making. The sample size of 135 patients, although sufficient to identify major trends, may limit the generalizability of the findings. Additionally, the study was conducted in a single tertiary care center, and results may differ in other institutions with varying patient populations, staffing models, or triage systems. Other limitations include the use of mixed triage systems (ESI and MTS), which could introduce variability in compliance classification, and the absence of linkage between triage accuracy and patient-centered outcomes.

Nevertheless, despite these limitations, the study provides valuable insights into real-world adherence to triage protocols. It highlights strengths, such as high compliance in critically ill patients, and weaknesses, particularly among stable patients and during peak hours. Future prospective multicenter studies with larger sample sizes are warranted to validate these findings and evaluate strategies to enhance triage compliance.

## Conclusions

Compliance with triage protocols in the ED was moderately high, with approximately three-fourths of assessments adhering to standardized guidelines. However, nearly one-fourth of cases were non-compliant, primarily due to under-triage, which poses serious risks to patient safety by potentially delaying critical interventions. Compliance was highest among critically ill patients but notably lower in stable cases and during peak hours, emphasizing the impact of patient load and systemic pressures on triage accuracy.

These findings underscore the need for targeted interventions to improve adherence to triage protocols across all acuity levels. Strategies such as routine staff training, simulated exercises to strengthen decision-making skills, regular audits with feedback, and the integration of digital triage support systems may enhance compliance, reduce variability, and ultimately improve patient safety and ED efficiency. Future research should focus on prospective, multicenter studies to evaluate these interventions and to develop evidence-based approaches to optimize triage practices across diverse clinical settings.
